# The Induction of Apoptosis in A375 Malignant Melanoma Cells by *Sutherlandia frutescens*

**DOI:** 10.1155/2016/4921067

**Published:** 2016-08-30

**Authors:** Nicola B. van der Walt, Zahra Zakeri, Marianne J. Cronjé

**Affiliations:** ^1^Department of Biochemistry, University of Johannesburg, P.O. Box 524, Auckland Park, Johannesburg 2006, South Africa; ^2^Department of Biology, Queens College, 65-30 Kissena Blvd., Flushing, NY 11367, USA

## Abstract

*Sutherlandia frutescens* is a medicinal plant indigenous to Southern Africa and is commonly known as the “cancer bush.” This plant has traditionally been used for the treatment of various ailments, although it is best known for its claims of activity against “internal” cancers. Here we report on its effect on melanoma cells. The aim of this study was to investigate whether an extract of* S. frutescens* could induce apoptosis in the A375 melanoma cell line and to outline the basic mechanism of action.* S. frutescens* extract induced apoptosis in A375 cells as evidenced by morphological features of apoptosis, phosphatidylserine exposure, nuclear condensation, caspase activation, and the release of cytochrome *c* from the mitochondria. Studies in the presence of a pan-caspase inhibitor allude to caspase-independent cell death, which appeared to be mediated by the apoptosis inducing factor. Taken together, the results of this study show that* S. frutescens* extract is effective in inducing apoptosis in malignant melanoma cells and indicates that further* in vivo* mechanistic studies may be warranted.

## 1. Introduction

Cancer is the leading cause of death in economically developed countries and the second leading cause of death in developing countries. In 2008, it was estimated that 12.7 million people around the world were suffering from cancer, while the disease led to the death of 7.6 million people [1]. In southern Africa the overall cancer incidence is given as 189.6 per 100 000 with a mortality rate of 133.2 per 100 000 [1]. Malignant melanoma is the 19th most common cancer worldwide [2] and the 10th most common cancer in South Africa [3]. Current treatment options include surgery, radiation therapy, chemotherapy, or a combination of these. However, all of these treatments have unpleasant and often harmful side effects. Thus, there remains a need for the identification of anticancer drugs with improved efficacy and fewer toxic side effects [4]. One source for such drugs is plants that are traditionally reputed to be effective against cancer.

*Sutherlandia frutescens* is a shrub indigenous to South Africa, Lesotho, Southern Namibia, and Southeastern Botswana. It was first used by the Khoi-San and early settlers in the Western Cape area as a general medicine to treat stomach complaints, internal cancers, wounds, and infections, and it is used by several cultural groups to this day [5]. However, the plant is best known for its reported anticancer activity and has locally been dubbed the “cancer bush.” It is commercially available in the form of tablets or tinctures.* Sutherlandia* SU1*™* tablets produced by Phyto Nova, South Africa, contain 300 mg* S. frutescens* subspecies* microphylla* (SU1) and have been used in previous studies [6–8]. Although batch-to-batch phytochemical analysis has not been reported, the major phytochemicals found in* S. frutescens* leaves (L-canavanine, D-pinitol, *γ*-aminobutyric acid, and sutherlandioside B [5, 9–11]) have also been identified in these tablets [8, 12].

The consumption of* S. frutescens* leaf powder is not associated with any toxic side effects. The Medical Research Council together with the National Research Foundation of South Africa investigated the consumption of* Sutherlandia* leaf powder in adult male vervet monkeys over a three-month period. Even at nine times the recommended dose (81.0 mg/kg) the monkeys did not display signs of any toxic or other side effects [13]. Furthermore, a randomized, double-blind, placebo-controlled trial in healthy human adults concluded that the consumption of 800 mg/d* Sutherlandia* leaf powder capsules for 3 months did not lead to any side effects and was tolerated well [14].

Previous studies have shown that aqueous and ethanolic extracts of* S. frutescens* have antiproliferative effects on various cancerous cell lines including breast cancer (MFC-7, MA-MB-231) [8, 15–17], cervical cancer (Caski) [18], esophageal cancer (SNO) [19], and leukemia cell lines (Jurkat and HL 60) [8] at concentrations ranging from 0.55 mg/mL to 10 mg/mL.* S. frutescens* extracts induce apoptotic-like morphology, externalization of phosphatidylserine (PS) molecules, increased caspase 3/7 activity, mitochondrial membrane depolarization, and expression of genes associated with apoptosis [6, 7, 16–19]. Therefore,* S. frutescens* appears to be able to induce apoptosis. Given that the effect of* S. frutescens* extract on melanoma is unknown, the aim of this study was to evaluate whether* S. frutescens* extract is able to induce apoptosis in A375 melanoma cells* in vitro* and to outline the basic mechanism of action.

## 2. Materials and Methods

### 2.1. Cell Culture

The A375 (malignant human melanoma) cell line was obtained from the European Collection of Cell Cultures. The human primary dermal fibroblast cell line (HDF*α*) was purchased from ScienCell, CA, USA. The Hek 293 (human embryonic kidney) cell line was a gift from Department of Plant Science at the University of Pretoria and the Colo-800 (human melanoma) cell line was a gift from the Institut für Molekulare Medizin at the Universitätsklinikum Düsseldorf. The A375 and Hek 293 cells were grown in Dulbecco's Modified Eagle Medium (DMEM) and the Colo-800 cells were grown in Roswell Park Memorial Institute (RPMI) medium. The base media was supplemented with 10% FBS, 1% penicillin/streptomycin/fungizone, and 1% gentamycin. The HDF*α* cells were cultured in fibroblast media supplemented with low serum growth supplement (ScienCell, CA, USA), 1% penicillin/streptomycin/fungizone, and 1% gentamycin. All cell lines were maintained at 37°C and 5% CO_2_ in a humidified atmosphere.

### 2.2. Preparation of* S. frutescens* Extracts

*Sutherlandia* SU1*™* tablets (Phyto Nova Natural Medicines, Isando, Gauteng, South Africa), each containing 300 mg* Sutherlandia frutescens* subspecies* microphylla* (SU1), were used in this study. UPLC-MS confirmed the identity of the plant material based on the presence of unique triterpenoids (sutherlandiosides) and flavonoids (sutherlandins). Ten tablets, containing a total of 3 g* S. frutescens*, were finely ground to a powder, added to 30 mL of 70% ethanol, and allowed to be extracted under constant agitation for 24 h. The suspension was centrifuged at 1028 ×g for 10 min. The supernatant was filtered twice through 0.22 *μ*m filters. The ethanol was evaporated and the residue was redissolved in sterile water to make stock solutions containing 100 mg residue per one mL water, which were again filtered through a 0.22 *μ*m filter and stored at −20°C.

### 2.3. Measuring Cell Viability in Response to* S. frutescens* Extract

Cells (5 × 10^3^) were seeded in 96-well plates and allowed to adhere for 24 h. The spent media was then discarded and replaced with new complete growth media, containing the appropriate concentration (0, 0.15, 0.3, 0.625, 1.25, or 2.5 mg/mL) of the* S. frutescens* extract. The cells were incubated at 37°C, 5% CO_2_ for the duration of the treatment period (24, 48, or 72 h).

To measure metabolism, a reflection of cell viability, at the end of the treatment period, 10 *μ*L alamarBlue® reagent (AbDSerotec, Kidlington, UK) was added to each well. The plate was incubated in the dark for 2 h at 37°C. Fluorescence was measured at an excitation wavelength of 544 nm and emission wavelength of 590 nm on the BioTek® Synergy*™*HT multidetection microplate reader (Winooski, VT, USA). The viability of the treated cells was expressed as a percentage relative to the control-treated cells (0 mg/mL).

### 2.4. Examination of Cellular Morphology following Treatment

Light microscopy was used to examine the gross morphology of cells. Following treatment with the vehicle control or 0.625 mg/mL* S. frutescens* extract, the cells were examined under the Carl Zeiss AxioCam MR color light microscope (Zeiss, Jena, Germany) at a 200x magnification to study the morphology of the cells. Micrographs of the cells were taken using AxioVision software.

### 2.5. Investigation of Apoptosis by Annexin V/PI Staining Flow Cytometry Assay

The extent of apoptosis was determined by flow cytometry using FITC-labelled annexin V and propidium iodide. Following treatment, the A375 cells were harvested and stained according to the instructions of the annexin V-FITC apoptosis detection kit 1 (BD Biosciences, San Diego, CA, USA). The cell samples were then analyzed on the FACS Aria*™* Flow Cytometer (BD Biosciences, San Diego, CA, USA). PI signal (emitted at 617 nm) was read on the PE-A channel while the annexin V-FITC signal (emitted at 530 nm) was read on the FITC-A channel. The data was obtained and analyzed using BD FACSDiva*™* 6.0 software.

### 2.6. Determination of Caspase Activity

To determine whether* S. frutescens* treatment resulted in caspase activation, the Caspase-Glo® 3/7, 8, and 9 assays from Promega (Madison, WI, USA) were used according to the manufacturer's instructions. The caspase activity of the treated cells was expressed as fold change compared to the control-treated cells.

### 2.7. Detection of Cleaved PARP by Western Blotting

Cells (1.2 × 10^6^) were seeded into 72 cm^2^ culture flasks and allowed to adhere for 24 h. They were treated for 24, 48, or 72 h. At the end of the treatment, the cells were collected and resuspended in 100 *μ*L cold 1x Laemmli extraction buffer (Bio-Rad, Hercules, CA, USA) containing *β*-mercaptoethanol (358 mM) and sonicated. The sample was then boiled for 10 minutes and centrifuged 16 000 ×g for 30 minutes. The supernatant was stored at −20°C. The protein samples (50 *μ*g) were separated on 10% polyacrylamide gels and transferred to Immun-Blot® 0.2 *μ*m PVDF membrane (Bio-Rad, Hercules, CA, USA). The membrane was blocked using 5% milk powder in 1x Tris-buffered saline (TBS) solution for an hour. The primary antibody in 5% milk powder solution (1 : 500 to 1 : 1000) was added to the membrane and incubated overnight at 4°C. The primary antibodies used were *β*-actin (Thermo Scientific, Rockford, IL, USA) and PARP (Abcam, Cambridge, UK). The membrane was washed three times with TBST (1x TBS with 0.1% Tween-20) for 5 minutes each. The horseradish peroxidase-linked secondary antibody anti-rabbit IgG (Sigma-Aldrich, St. Louis, MO, USA) and anti-mouse IgG (Thermo Scientific, Rockford, IL, USA) in 2.5% milk (1 : 5000) were added and incubated for 1 h. The membrane was washed four times with TBST solution for 5 minutes each. The chemiluminescent substrate was made fresh and added to the membrane for 5 min under constant agitation. Excess solution was drained and the blot was imaged using the ChemiDoc MP System (Bio-Rad, Hercules, CA, USA).

### 2.8. Immunofluorescence Microscopy for the Detection of Cytochrome *c* Release

Following treatment, growth medium containing 100 nM MitoTracker probe® (Molecular Probes, Eugene, OR, USA) was added to the culture, which was incubated for 30 min at 37°C. The cells were washed with PBS and then fixed in 4% formaldehyde in PBS for 15 min at 37°C. After washing the cells, they were permeabilized with 0.1% Triton X-100 in PBS for 15 min at 4°C. To prevent nonspecific binding, the cells were incubated in blocking buffer (1% BSA in PBST, pH 7.4) for 30 min at room temperature. Anti-cytochrome *c* antibody (0.5 *μ*g/mL; 2 mL) (Abcam, Cambridge, MA, USA) was added and incubated for 1 h at room temperature. The coverslip was washed and then incubated in 2 mL Hoechst 33258 stain (1 *μ*g/mL in PBS) (Sigma-Aldrich, St. Louis, MO, USA) for 20 minutes. The coverslip was washed again before being mounted with a drop of mounting medium (90% glycerol in PBS) and sealed with nail polish and stored in the dark at 4°C. The slide was examined on the Axioplan 2 fluorescence microscope (Zeiss, Jena, Germany) at 630x magnification and micrographs of the cells were taken using AxioVision 4.8 software. The excitation and emission wavelengths (nm) of the fluorescent dyes were as follows: Alexa-488, excitation: 499 and emission: 519; MitoTracker orange probe, excitation: 554 and emission: 576; and Hoechst 33258, excitation: 343 and emission: 483.

### 2.9. Cell Death in the Presence of Caspase Inhibitors

To reveal any underlying caspase-independent pathways induced by* S. frutescens* extract, Z-VAD-fmk (Sigma-Aldrich, St. Louis, MO, USA), an irreversible pan-caspase inhibitor, was added to the cells prior to treatment. Cells were pretreated with 20 *μ*M Z-VAD-fmk in DMSO for 1 h to inhibit caspases and then treated with* S. frutescens* extract. Cell viability, morphology, and PS externalization were investigated as previously described.

### 2.10. Determining the Subcellular Location of the Apoptosis Inducing Factor (AIF) Using Immunofluorescence Microscopy

Following treatment, the cells were washed with 1 mL of prewarmed PBS and then fixed in 4% formaldehyde in PBS for 15 min at room temperature. The coverslip was washed in 1x PBS and then incubated in 2% Triton X-100 in PBS for 15 min at room temperature. To prevent nonspecific binding the coverslip was incubated in blocking buffer (3% BSA, 1% Triton X-100 in PBS) for 1 h at room temperature. Anti-AIF antibody (0.5 *μ*g/mL; 2 mL) (Abcam, Cambridge, MA, USA) was added and the mixture was incubated for 4°C overnight. The coverslip was washed before incubating in 2 mL donkey anti-rabbit IgG (1 : 1000) (Abcam, Cambridge, MA, USA) for 1 h at room temperature. After washing the coverslip, it was incubated in Hoechst 33258 stain and prepared and analyzed as described above.

### 2.11. Statistical Analyses

Three independent experiments with two or three technical replicates of each of the various assays were performed. The data relevant to a particular assay was presented in bar graphs where the error bars were used to represent the standard error of the mean (SEM) for each condition. To determine whether there were significant differences between the treated and untreated cells, two-tailed Student's *t*-test was performed in Microsoft® Excel. If the probability value (*P* value) was less than 0.05, it was concluded that the values for the treated cells were significantly different with respect to the control-treated cells.

## 3. Results

### 3.1. *S. frutescens* Extract Reduces Cell Viability in a Time- and Dose-Responsive Manner

**Figure 1:**
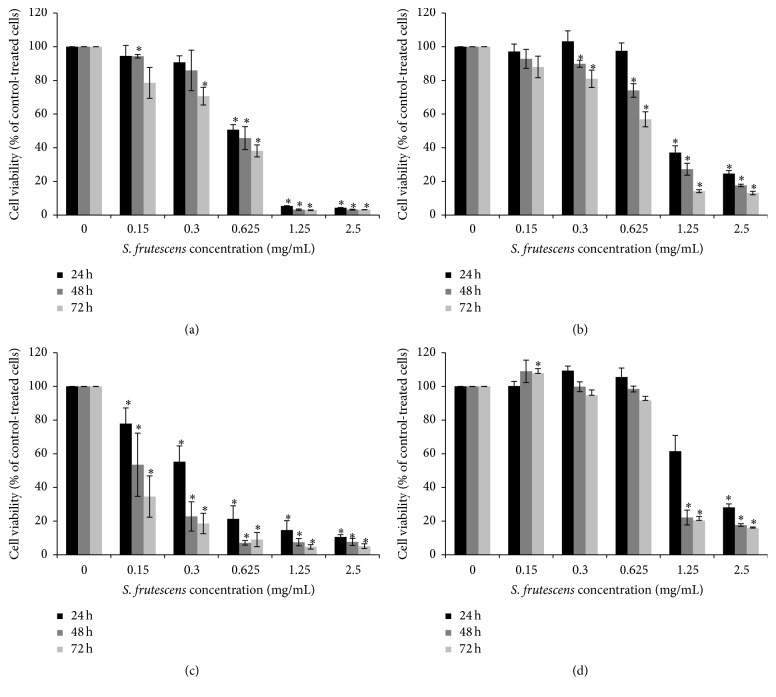
The effect of* S. frutescens* extract on the viability of (a) A375 melanoma, (b) Colo-800 melanoma, (c) HDF*α* fibroblast, and (d) Hek 293 cells after 24, 48, and 72 h of treatment. The viability was assessed using the alamarBlue assay and expressed as percentage relative to the control-treated cells (0 mg/mL). Error bars represent the SEM (*n* ≥ 3) and *∗* indicates a significant difference from 0 mg/mL (*P* < 0.05).

To determine the cytotoxicity of* S. frutescens* extract, time- and dose-response studies were conducted using the A375 and Colo-800 melanoma as well as HDF*α* and Hek 293 normal cell lines. As seen in Figure 1, the* S. frutescens* extract was cytotoxic to both melanoma cell lines and caused a general dose- and time-dependent decrease in cell viability. When A375 cells were treated with the extract at a concentration of 0.625 mg/mL for 24 h, the viability was 51% compared to control-treated cells (Figure 1(a)). This decreased to 46% and 38% after 48 h and 72 h of treatment, respectively. The Colo-800 cells were more resistant to the extract and the viability was 98% compared to the control after 24 h of treatment (Figure 1(b)). This decreased to 74 and 57%, compared to the control-treated cells, after 48 and 72 h of treatment, respectively.

Interestingly, the normal HDF*α* cell line was extremely sensitive to* S. frutescens* extract (Figure 1(c)). Treatment with* S. frutescens* extract at a concentration of 0.3 mg/mL reduced the viability of the HDF*α* cells to approximately 55% compared to control-treated cells after 24 h. After 72 h of treatment with 0.3 mg/mL* S. frutescens* extract, the viability of HDF*α* cells was just 19%. On the other hand, the normal Hek 293 cells were the least sensitive to the* S. frutescens* extract (Figure 1(d)). At a concentration of 0.625 mg/mL, the extract had a slight, but not significant, proliferative effect on these cells after 24 and 48 h of treatment. When the treatment time was increased to 72 h, the viability of the Hek 293 cells was 90% compared to control-treated cells.

For the remainder of this study we focused on the effects of* S. frutescens* extract on the A375 malignant melanoma cells, since these were the most responsive of the melanoma cells tested. A concentration of 0.625 mg/mL* S. frutescens* extract was selected for further studies because it was the lowest concentration that significantly reduced the A375 cell viability after 24 h of treatment. Three time points (24, 48, and 72 h) were used to study the progression of cell death in response to the extract.

### 3.2. Morphological Changes Are Observed in Response to* S. frutescens* Extract

**Figure 2:**
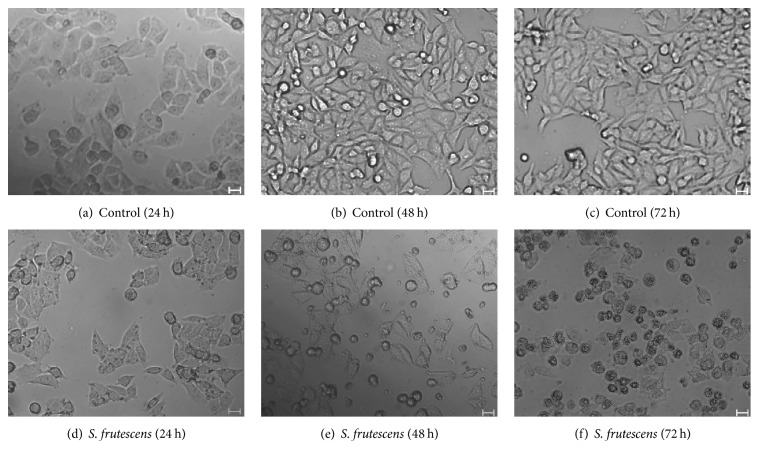
The effect of* S. frutescens* on the morphology of A375 cells. Control-treated cells (a)–(c) are typical, healthy A375 cells which become more confluent with an increase in treatment time.* S. frutescens* extract causes morphological changes in the A375 cells (d)–(f). These include the loss of adherence and cell shrinkage. The scale bar represents 20 *μ*m.

The control-treated cells exhibited typical, healthy morphology (Figures 2(a)–2(c)). The cells were spindle-shaped and adhered well to the cell culture dish. As the treatment time increased, the cells became more confluent. After 24 h of treatment with* S. frutescens*, the cells did not appear morphologically different from the control-treated cells (Figure 2(d)), although they were less confluent and a few cells had detached from the cell culture dish. As the treatment time was increased to 48 and 72 h, considerably more detached cells were observed and they appeared slightly shrunken, compared to the adherent cells (Figures 2(e) and 2(f)).

### 3.3. Externalization of Phosphatidylserine Molecules in Response to* S. frutescens* Treatment Indicates the Induction of Apoptosis

**Figure 3:**
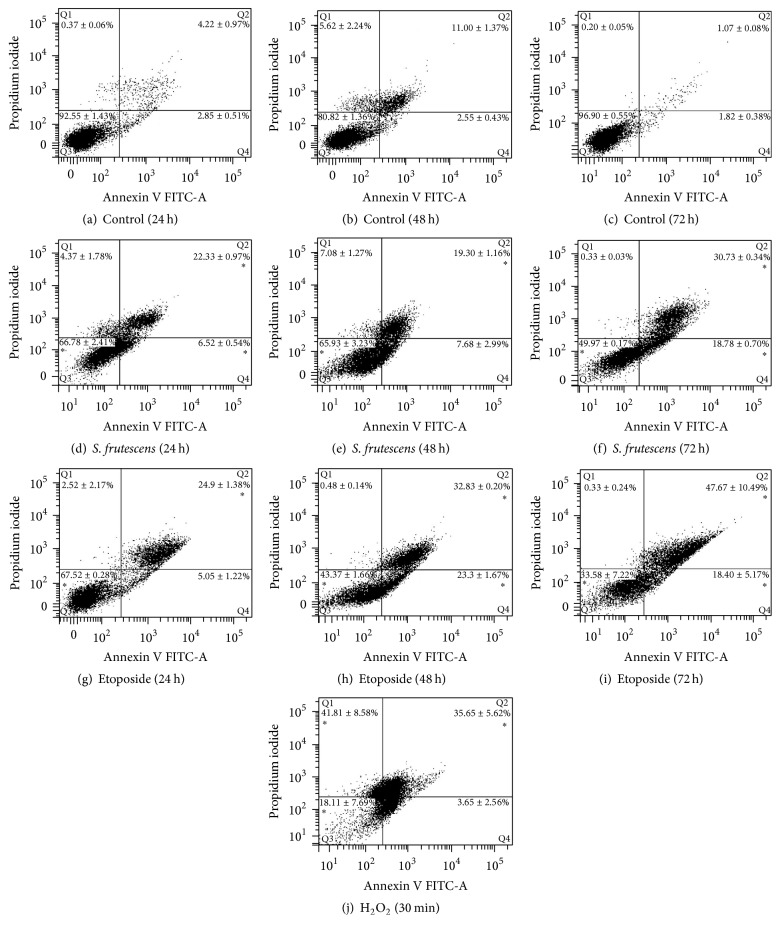
*S. frutescens* extract induces apoptosis in A375 cells. Representative scatterplots of annexin V-FITC and PI for control-treated cells (a)–(c), cells treated with* S. frutescens* (d)–(f), cells treated with etoposide, and a known inducer of apoptosis (g)–(i) for 24, 48, and 72 h as well as cells treated with hydrogen peroxide for 30 min, which served as a necrotic control (j). The percentage of the cell population ± the SEM (*n* = 3) that is in Q1 (necrotic), Q2 (late apoptotic), Q3 (viable), or Q4 (early apoptotic) is shown. A significant difference compared to the control-treated cells is indicated by *∗* (*P* < 0.05).

To determine whether* S. frutescens* extract induced apoptosis in the A375 cells, the translocation of phosphatidylserine (PS) from the inner to the outer leaflet of the plasma membrane was studied. As seen in Figures 3(a)–3(c), the majority of the control-treated cells were healthy and unstained by either FITC-labelled annexin V or PI at all three treatment times.* S. frutescens* treatment resulted in the translocation of PS molecules, as evidenced by the binding of FITC-labelled annexin V (Figures 3(d)–3(f)). Following treatment with* S. frutescens* extract for 24 h, approximately 7 and 22% of cell population were in the early stages (annexin V^+^/PI^−^) and late stages of apoptosis (annexin V^+^/PI^+^), respectively. This increased to approximately 19 and 31%, respectively, after 72 h of treatment (Figure 3(f)). Etoposide-treated cells served as positive apoptotic controls. As seen in Figures 3(g)–3(i), PS translocation was observed in response to etoposide with approximately 5 and 25% of cell population in the early and late stages of apoptosis, respectively, after 24 h treatment. As the treatment time increased to 72 h, so the early and late apoptotic populations increased to approximately 19 and 48%, respectively. Cells treated with H_2_O_2_ served as a control for necrosis (Figure 3(j)) with approximately 42% of the cell population in the necrosis quadrant (annexin V^−^/PI^+^). These results show that* S. frutescens* extract and etoposide induce apoptosis while H_2_O_2_ induces necrosis.

### 3.4. The Release of Cytochrome *c* Suggests the Involvement of the Mitochondrial Apoptotic Pathway

**Figure 4:**
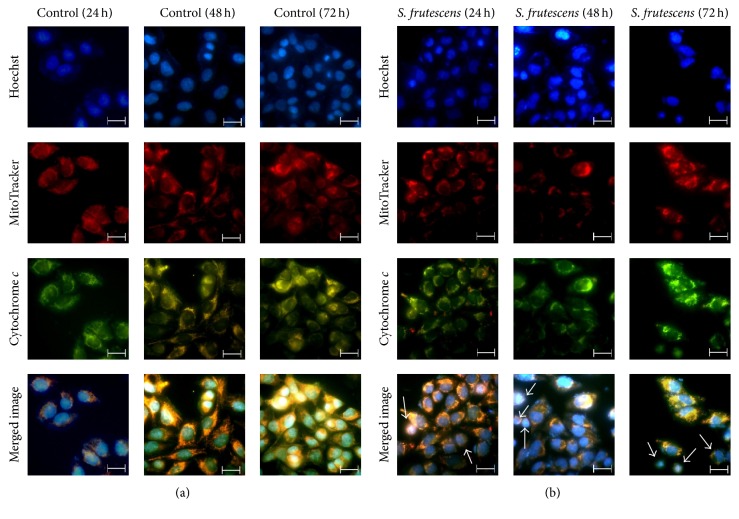
Cytochrome *c* is released into the cytoplasm in response to* S. frutescens* treatment. Control and* S. frutescens*-treated cells were stained with Hoechst 33258, which binds to DNA, MitoTracker orange, which accumulates in active mitochondria, and Alexa-488-tagged anti-cytochrome *c* antibody, which labels cytochrome* c*. (a) The control-treated cells have intact mitochondrial membrane potential and cytochrome *c* which is localized in the mitochondria. (b) In the* S. frutescens*-treated cells, the cytochrome *c* staining appeared more diffuse and less intense yellow staining was observed in the merged image as indicated by white arrows. This is due to a reduced overlap of cytochrome *c* and MitoTracker orange and indicates the release of cytochrome *c* from the mitochondria. The scale bar represents 20 *μ*m.

The induction of apoptosis by many anticancer drugs involves the release of cytochrome *c* from the mitochondria [20]. Therefore, immunofluorescence was used to determine whether cytochrome *c* was released from the mitochondria in response to* S. frutescens* (Figure 4). The control-treated cells had healthy nuclear morphology as revealed by the uniform distribution of the Hoechst 33258 stain in the nuclei (Figure 4(a), top row). The MitoTracker orange accumulated in the active mitochondria and bright orange-red staining was observed (Figure 4(a), second row). The cytochrome *c* staining was bright green and concentrated in the mitochondria (Figure 4(a), third row). The colocalization of the MitoTracker and cytochrome *c* in the mitochondria was indicated by bright yellow staining (Figure 4(a), bottom row).

The nuclei of many of the cells treated with* S. frutescens* extract were condensed and fragmented into apoptotic bodies (Figure 4(b), top row). The cytochrome *c* staining was less intense and more diffuse than that of the control-treated cells (the color differences in the prints derive from differences in photobleaching). There was less overlap of the MitoTracker and cytochrome *c* staining, indicated by the reduced yellow staining in the merged image (Figure 4(b), bottom row). This result, best visualized in the merged image, suggests that cytochrome *c* was released from the mitochondria.

### 3.5. Caspases Are Activated following* S. frutescens* Treatment

**Figure 5:**
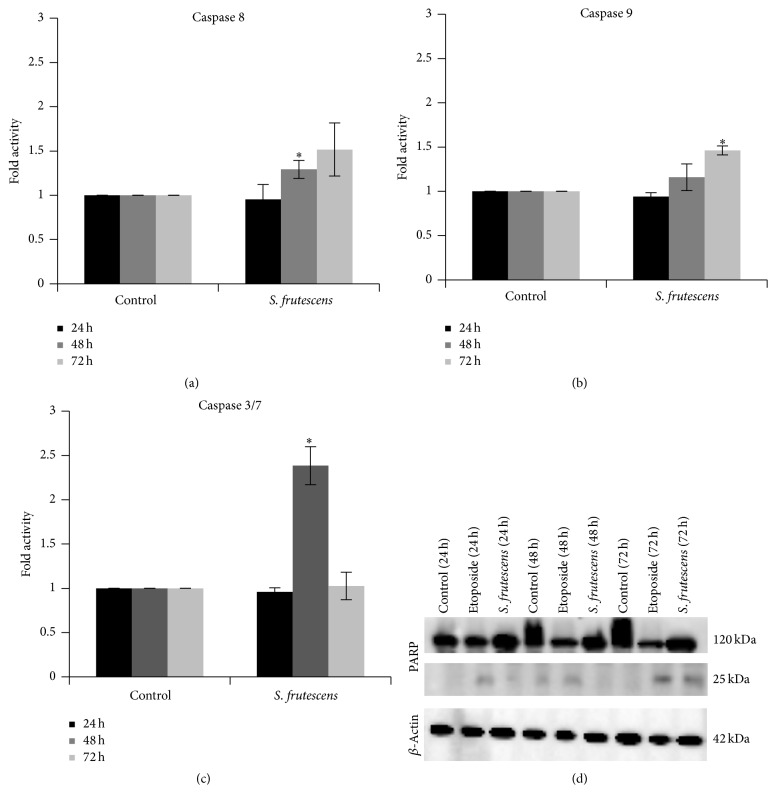
*S. frutescens* extract causes caspase activation. The activity of (a) caspase 8, (b) caspase 9, and (c) caspase 3/7 in control- and* S. frutescens*-treated A375 cells was measured using the Caspase-Glo assays 24, 48, and 72 h after treatment. The activity of the caspases in the* S. frutescens*-treated cells is shown as a fold increase relative to the control-treated cells. Error bars represent the SEM (*n* = 3) and *∗* indicates a significant difference compared to the control-treated cells (*P* < 0.05). (d) Cleaved PARP was detected by western blot and confirmed the activity of the executioner caspases in response to* S. frutescens* treatment. Etoposide treatment served as a positive apoptotic control and *β*-actin served as a loading control.

Apoptosis is often associated with an increase in caspase activity. To determine whether* S. frutescens* extract-induced cell death was caspase-dependent, the activity of caspases 8, 9, and 3/7 was investigated 24, 48, and 72 h after treatment and compared to control-treated cells. The activity of caspases 8, 9, and 3/7 for the control-treated cells was set to 1.00, as they served as a reference for the* S. frutescens*-treated cells (Figure 5).

No significant increases in caspase activity were observed 24 h after treatment with* S. frutescens* extract. By 48 h a slight, but significant, 1.29-fold increase in caspase 8 activity was observed compared to the control-treated cells (Figure 5(a)). The activity of caspase 3/7 also increased significantly (2.38-fold) compared to the control-treated cells after 48 h of treatment (Figure 5(c)). When the treatment time was increased to 72 h, a significant 1.46-fold increase in the activity of caspase 9 was observed compared to the control-treated cells (Figure 5(b)).

PARP is a substrate for the executioner caspases and its cleavage can indirectly be proof of executioner caspase activation. Full length PARP (120 kDa) was detected by western blot in the lysates of all cells after 24, 48, or 72 h of treatment (Figure 5(d)). PARP was cleaved to produce a 25 kDa fragment in response to* S. frutescens* extract after 72 h of treatment. This cleaved PARP fragment was also produced in response to etoposide, a positive apoptotic control, after 24, 48, and 72 h of treatment. This confirms the activity of the executioner caspase 3 and/or caspase 7 following* S. frutescens* treatment.

### 3.6. *S. frutescens* Extract Induces Cell Death in the Presence of a Pan-Caspase Inhibitor

Caspase inhibition studies were performed to examine the role of the caspases in* S. frutescens*-induced cell death further. Cells were pretreated with 20 *μ*M Z-VAD-fmk, an irreversible pan-caspase inhibitor, before being treated with* S. frutescens* extract as per previous experiments.

**Figure 6:**
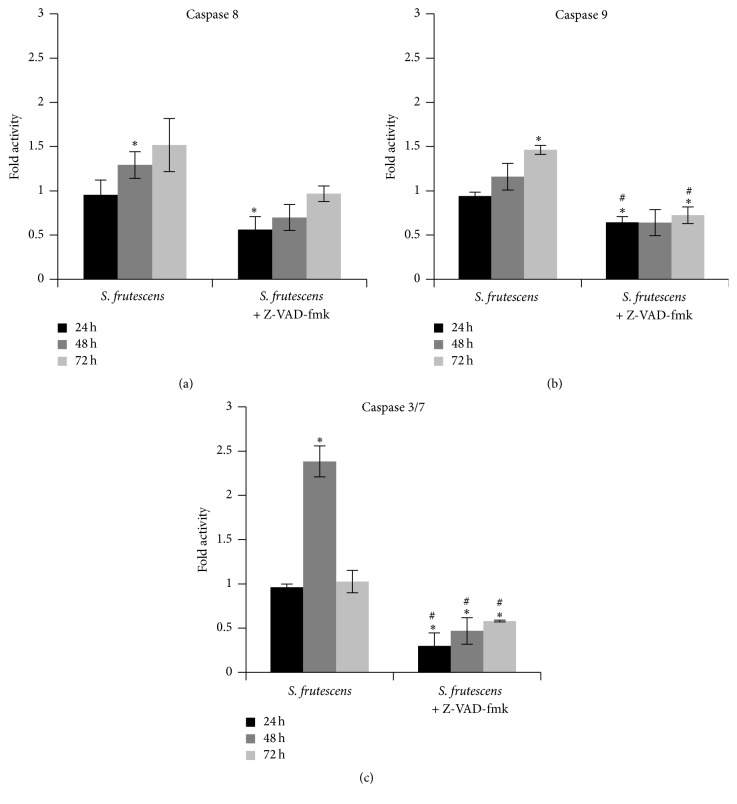
Z-VAD-fmk prevents caspase activation in response to* S. frutescens* extract. The activity of (a) caspase8, (b) caspase 9, and (c) caspase 3/7 was measured in cells treated with* S. frutescens* extract in the absence and presence of Z-VAD-fmk for 24 h, 48 h, and 72 h and expressed as a fold increase relative to the control cells. Error bars represent the SEM (*n* = 3), *∗* indicates a significant difference from the control-treated cells (*P* < 0.05), and # indicates a significant difference from the cells treated with* S. frutescens* alone.

**Figure 7:**
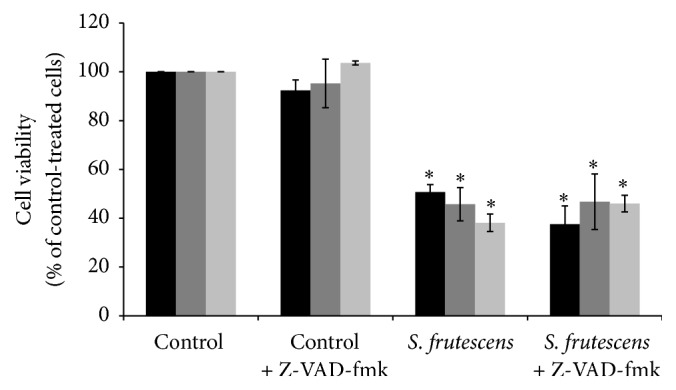
Z-VAD-fmk did not prevent the reduction of cell viability in response to* S. frutescens* extract. Cell viability was assessed using the alamarBlue assay and expressed as percentage relative to the control-treated cells. Error bars represent the SEM (*n* = 3) and *∗* indicates a significant difference from the control-treated cells (*P* < 0.05). No statistical differences were observed in the viability of the cells treated with* S. frutescens* in the presence or absence of Z-VAD-fmk.

In all instances, Z-VAD-fmk successfully inhibited the activation of caspases 8, 9, and 3/7 in response to* S. frutescens* extract (Figures 6(a)–6(c)). No statistical difference in the viability of the control-treated cells in the presence of Z-VAD-fmk was observed when compared to those cells not treated with the inhibitor (Figure 7), indicating that Z-VAD-fmk is not toxic to the cells. Despite the caspase inhibition, by Z-VAD-fmk,* S. frutescens* extract significantly reduced the cell viability when compared to the control-treated cells (Figure 7). However, there was no statistical difference in the viability of the cells treated with* S. frutescens* extract in the presence or absence of Z-VAD-fmk at any time (24, 48, or 72 h). This likely indicates that in the event of caspase inhibition* S. frutescens* extracts are able to induce cell death in melanoma cells.

**Figure 8:**
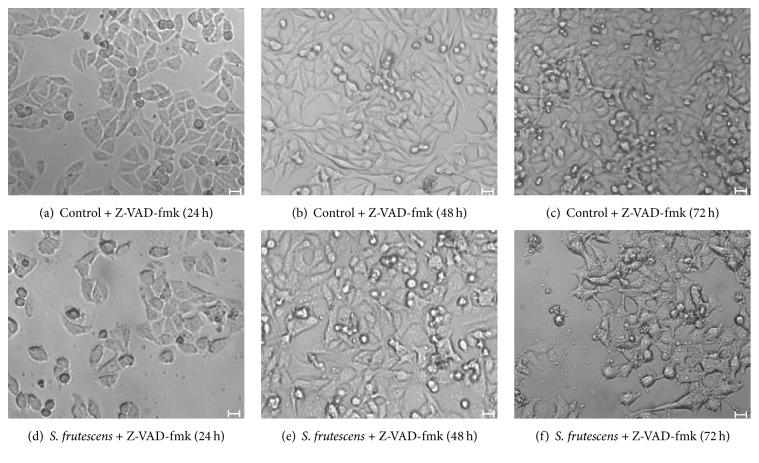
Morphology of A375 cells, pretreated with Z-VAD-fmk, in response to control or* S. frutescens* treatment after 24, 48, or 72 h. The pretreatment with Z-VAD-fmk did not alter the morphology of the control-treated cells (a)–(c) indicating that it is not cytotoxic. Z-VAD-fmk did not prevent cell detachment in response to* S. frutescens* extract (d)–(f). Scale bar represents 20 *μ*m.

The addition of Z-VAD-fmk alone did not have a detrimental effect on the morphology of the A375 cells and healthy cellular morphology of the control-treated cells was observed in the presence (Figures 8(a)–8(c)) and absence (Figures 2(a)–2(c)) of Z-VAD-fmk. Cell detachment in response to* S. frutescens* extract was not prevented by Z-VAD-fmk (Figures 8(d)–8(f)).

**Figure 9:**
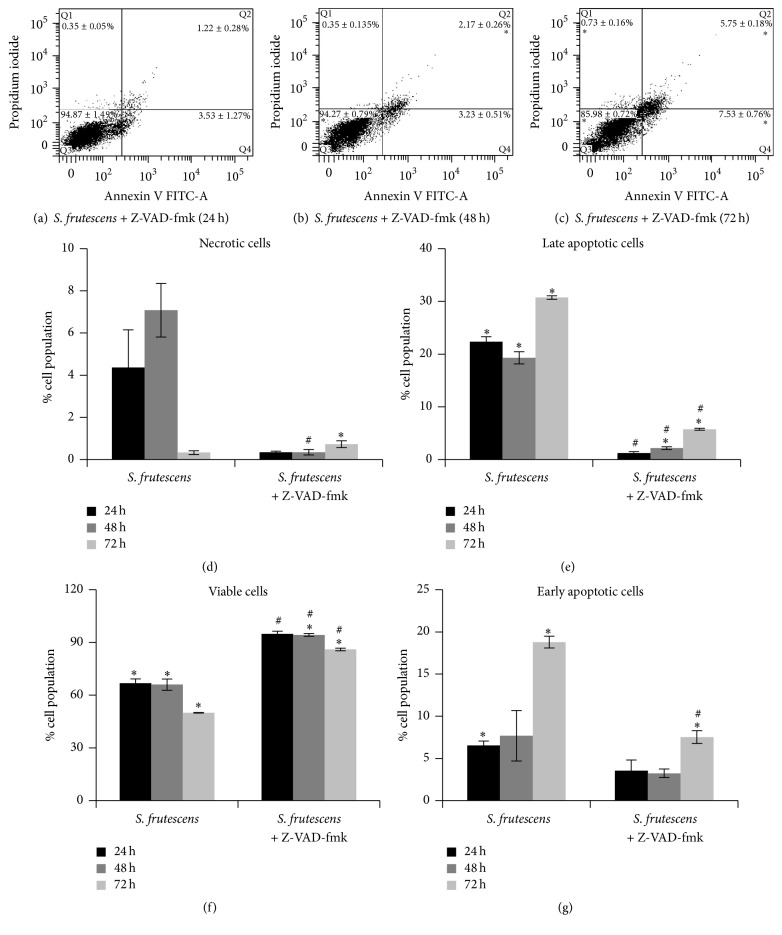
Z-VAD-fmk prevents the externalization of PS following* S. frutescens* treatment. Representative scatterplots of FITC-labelled annexin V and PI for cells pretreated with 20 *μ*M Z-VAD-fmk followed by treatment with* S. frutescens* for (a) 24 h, (b) 48 h, and (c) 72 h. The percentage of the cell population ± the SEM (*n* = 3) that is in Q1 (necrotic), Q2 (late apoptotic), Q3 (viable), or Q4 (early apoptotic) is shown on the scatterplots. A comparison between the percentages of the cell population ± the SEM (*n* = 3) that is (d) necrotic, (e) late apoptotic, (f) viable, or (g) early apoptotic in cells treated with* S. frutescens* in the absence or presence of Z-VAD-fmk; *∗* indicates a significant difference compared to the control-treated cells; # indicates a significant difference compared to the cells treated with* S. frutescens* alone (*P* < 0.05).

Z-VAD-fmk blocked the exposure of PS molecules in response to* S. frutescens* treatment (Figure 9). The majority (>85%) of cells treated with* S. frutescens* in the presence of Z-VAD-fmk were negative for both FITC and PI and therefore accumulated in the viable quadrant (Q3). Small early and late apoptotic populations of approximately 1–4% were observed after 24 h (Figure 9(a)) and 48 h (Figure 9(b)) of treatment with* S. frutescens* in the presence of Z-VAD-fmk, increasing to approximately 6 to 8% after 72 h (Figure 9(c)) of treatment in the presence of Z-VAD-fmk. The early and late apoptotic populations were significantly smaller when cells were treated with* S. frutescens* extract in the presence of Z-VAD-fmk than those treated with* S. frutescens* extract alone (Figures 9(e) and 9(g)). The viable cell population was significantly larger when cells were treated with* S. frutescens* extract in the presence of Z-VAD-fmk than those treated with* S. frutescens* extract alone (Figure 9(f)).

### 3.7. AIF Translocation May Be Involved in the Caspase-Independent Cell Death Pathway Induced by* S. frutescens* Extract

**Figure 10:**
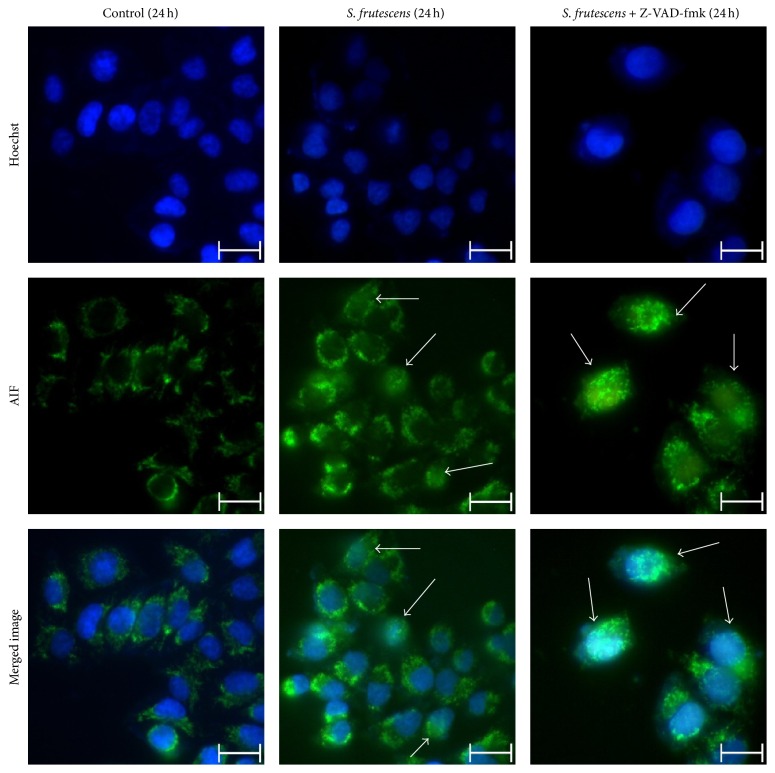
AIF translocates to the nucleus in response to* S. frutescens* extract. AIF is localized in the mitochondria of control-treated cells.* S. frutescens* treatment induces the nuclear translocation of AIF, as indicated by white arrows. Z-VAD-fmk does not prevent the nuclear translocation of AIF in response to* S. frutescens*. Cells were stained with Hoechst 33258 to label the nuclei and Alexa-tagged anti-AIF antibody to determine the subcellular localization of AIF. The white arrows indicate the nuclear localization of AIF. The scale bar represents 20 *μ*m.

Caspase-independent cell death is often mediated by AIF, which translocates from the mitochondria to the nucleus. The subcellular localization of AIF was studied using immunofluorescence microscopy (Figure 10) to determine whether it played a role in the caspase-independent apoptotic pathway induced by* S. frutescens*. The time point of 24 h was selected for this investigation because the activity of the caspases was lowest when the cells were treated with* S. frutescens* in the presence of Z-VAD-fmk compared to* S. frutescens* alone at this time.

In the control-treated cells, AIF was localized in the mitochondria (Figure 10, left panel). Following treatment with* S. frutescens*, AIF translocated to the nucleus as evidenced by the colocalization of the Alexa-488-tagged-anti-AIF with the Hoechst 33258 stain (Figure 10, center panel). The inhibition of caspases by Z-VAD-fmk prior to treatment with* S. frutescens* did not affect AIF nuclear translocation (Figure 10, right panel). Taken together, this data suggests that AIF translocation may play a role in the caspase-independent cell death pathway induced by* S. frutescens*.

## 4. Discussion

We aimed to determine whether an extract of* S. frutescens* could induce apoptosis in melanoma cells* in vitro* and to outline the basic mechanism of action. The results showed that* S. frutescens* extract was cytotoxic to melanoma cell lines in a dose- and time-dependent manner. Further analysis revealed the induction of both caspase-dependent and caspase-independent cell death in A375 melanoma cells treated with the plant extract.

*S. frutescens* has been dubbed the “cancer bush” in South Africa due to its reported anticancer activities. Previous studies have found that extracts of the plant have antiproliferative effects on various cancerous cell lines including breast cancer (MFC-7, MA-MB-231), cervical cancer (Caski), oesophageal cancer (SNO), and leukaemia cell lines (Jurkat and HL 60) [8, 16–19]. We report here that* S. frutescens* extract reduced the viability of A375 as well as Colo-800 human melanoma cells in a dose- and time-dependent manner. The A375 cells were more sensitive to the extract and therefore this cell line was selected for further experimentation. The concentration of 0.625 mg/mL was selected for further experiments since it reduced the viability to approximately 50% compared to the control-treated cells after 24 h.

The reduction of cell viability in response to* S. frutescens* extract was due to the induction of apoptosis which was evidenced by the externalization of PS molecules, nuclear condensation and fragmentation, the activation of caspases 8, 9, and 3/7, the translocation of cytochrome *c* from the mitochondria to the cytoplasm, and cleavage of PARP. Furthermore, in a Cancer Pathway Finder RT^2^ PCR array study (SA Biosciences) (data not shown) significant increases in the expression of the CASP9 and FASLG genes in response to* S. frutescens* extract indicate that the extract may induce apoptosis via the intrinsic as well as extrinsic pathways.

Although caspase-dependent apoptosis is often cited as the molecular mechanism by which cancer chemotherapies exert their antitumor effects, there is increasing evidence of anticancer compounds inducing cell death that is completely independent of caspase activation. According to Leist and Jäättelä (2001) the inhibition of caspase activation may reveal underlying caspase-independent cell death pathways [21]. Chipuk and Green (2005) define caspase-independent cell death as “the loss of cell viability that is induced by pro-apoptotic conditions, and which proceeds despite the inhibition or disruption of caspase function” [22]. To reveal any underlying caspase-independent cell death pathways induced by* S. frutescens* extract, the pharmacological, irreversible pan-caspase inhibitor Z-VAD-fmk was used. The externalization of PS molecules in response to* S. frutescens* treatment was blocked by Z-VAD-fmk pretreatment since it is reported to be largely caspase-dependent [23]. Similarly, previous studies have reported that Z-VAD-fmk inhibited PS externalization in evodiamine-treated U937 (leukemia) cells [24], actinomycin D-treated K562 (erythroleukemia) cells [25], and Bel-7402 (hepatocellular carcinoma) cell treated with* Ligustrum lucidum* fruit extract [26].

Interestingly, pretreatment with Z-VAD-fmk prevented neither cell detachment nor reduction of cell viability in response to* S. frutescens* extract, indicating the involvement of caspase-independent cell death. AIF translocated into nucleus following* S. frutescens* treatment and this translocation was not inhibited by pretreatment with Z-VAD-fmk, indicating that the caspase-independent pathway induced by* S. frutescens* may be mediated by AIF. Similar AIF-mediated caspase-independent pathways have been reported in response to evodiamine, staurosporine, and matrine, which are all plant-derived drugs [24, 27, 28]. Such caspase-independent cell death pathways are important defense mechanisms should the apoptotic pathway be evaded in cancer cells [21, 29].

The effect of* S. frutescens* extract on normal cells was also studied. Of the four cell lines studies, the Hek 293 cells were the least sensitive to* S. frutescens* treatment while the normal epithelial HDF*α* cells were the most sensitive. Similarly, Phulukdaree et al. (2010) have also reported that an ethanolic extract of* Sutherlandia* tablets from Phyto Nova did not significantly affect the viability of MDBK and LLC-PKI kidney cells at a concentration of 0.6 mg/mL [7]. We have previously studied the relative sensitivity of A375, HDF*α*, and Hek 293 cells in response to cisplatin, a known apoptotic inducer. We found that A375 cells were the most sensitive to 100 *μ*M cisplatin treatment, followed by HDF*α* cells with the Hek 293 cells being the least sensitive [30]. It was therefore surprising to find out that the HDF*α* cells were more sensitive to* S. frutescens* extract than the A375 cells, raising concerns about the potential toxicity of the extract. Previous studies have shown that although* S. frutescens* extracts have been shown to exert antiproliferative effects on normal cells such as breast (MCF12A) and blood (PBMC) cells, the extracts were more toxic to cancerous cells than to the noncancerous cells even at concentrations of up to 10 mg/mL [8, 16–19]. Furthermore,* in vivo* studies in both vervet monkeys and humans reported that the consumption of* S. frutescens* leaf powder was not associated with any toxic side effects even at nine times the recommended dose (81.0 mg/kg) [13, 14]. In fact, given that the concentration of* S. frutescens* required to reduce the viability in any cell line is quite high indicates that the extract is not highly toxic. This brings into question the physiological relevance of the extract for cancer treatment.

In ethnopharmacology, a wide range of crude extract concentrations are used for* in vitro* studies and there are no generally accepted guidelines for antiproliferative or cytotoxic IC_50_ concentrations. Gertsch (2009) cautions that “crude extract concentrations above 200 *μ*g/mL are likely to be artificial despite yielding reproducible effects” [31]. However, it is often difficult to correlate* in vitro* findings to an* in vivo* situation. There may differences in efficacy and toxicity* in vitro* and* in vivo* due to the metabolism, bioavailability, and pharmacokinetics of* S. frutescens* following oral consumption. This highlights the need for* in vivo* pharmacokinetic and mechanistic studies of* S. frutescens*, especially in a cancer model, in the future.

## 5. Conclusion

In conclusion,* S. frutescens* extract exerts a cytotoxic effect on melanoma cells that involves the induction of apoptosis. The extract was able to induce extrinsic and intrinsic caspase-dependent apoptosis as well as AIF-mediated caspase-independent cell death. The simultaneous activation of multiple cell death pathways by* S. frutescens* extract maximizes its anticancer effect. Furthermore, gene analysis (data not shown here) revealed several new molecular targets of* S. frutescens* extract. These include angiogenesis, metastasis, EMT (epithelial-to-mesenchymal transformation), hypoxia, cell cycle regulation, and cell senescence. However, the relatively high concentration of the extract required to elicit such a response may pose a significant limitation for the clinical use of* S. frutescens* for the treatment of cancer.
